# Removal of a large symptomatic retrocardiac mediastinal lipoma

**DOI:** 10.1093/jscr/rjae273

**Published:** 2024-05-04

**Authors:** Martina Wollheim, Lily F S Willatt, Jonas P Ehrsam, Priska Cerncic, Mario L Lachat, Othmar Schöb, Ilhan Inci

**Affiliations:** University of Nicosia, Medical School, Nicholas St 93, Egkomi Lefkosias 2408, Nicosia, Cyprus; Surgery, Klinik Hirslanden Zürich, Chirurgisches Zentrum, Witellikerstrasse 40, 8032 Zurich, Switzerland; University of Nicosia, Medical School, Nicholas St 93, Egkomi Lefkosias 2408, Nicosia, Cyprus; Surgery, Klinik Hirslanden Zürich, Chirurgisches Zentrum, Witellikerstrasse 40, 8032 Zurich, Switzerland; Surgery, Klinik Hirslanden Zürich, Chirurgisches Zentrum, Witellikerstrasse 40, 8032 Zurich, Switzerland; Institute for Histology and Cytology Diagnosis, AG, Dammweg 1, 5000 Aarau, Switzerland; Vascular Surgery, Klinik Hirslanden Zürich, Aorten und Gefässzentrum - Witellikerstrasse 40, 8032 Zürich, Switzerland; University of Zürich, Rämistrasse 71, 8006 Zürich, Switzerland; Surgery, Klinik Hirslanden Zürich, Chirurgisches Zentrum, Witellikerstrasse 40, 8032 Zurich, Switzerland; University of Zürich, Rämistrasse 71, 8006 Zürich, Switzerland; Surgery, Klinik Hirslanden Zürich, Chirurgisches Zentrum, Witellikerstrasse 40, 8032 Zurich, Switzerland; University of Zürich, Rämistrasse 71, 8006 Zürich, Switzerland

**Keywords:** retrocardiac lipoma, surgical resection, mediastinal lesion, soft-tissue tumor, case report

## Abstract

Large mediastinal lipomas are rare. Complete surgical resection can be difficult due to the intricate anatomy in the mediastinum. We report the case of a 75-year-old man with worsened retrosternal pressure, decline in performance and syncope episodes. Computed tomography revealed a large retrocardiac low-attenuated mediastinal lesion measuring 10 × 8 cm, compressing the left atrium and pulmonary veins bilaterally. Surgical exploration was achieved through a right anterolateral thoracotomy with a successful en bloc resection without any intraoperative complications. The total operation time was 185 min with a total blood loss of <250 ml. Stand-by extracorporeal life support was present throughout the procedure, but its use was not required. The postoperative course was uneventful. The pathological examination revealed a mature mediastinal lipoma without any evidence of malignancy. In the 12-month control the patient was completely free of symptoms and in a good general condition.

## Introduction

Lipomas are well-demarcated, slow-growing mesenchymal tumors originating from adipose tissue, predominantly manifesting a benign entity. Mediastinal location is rare and are mostly incidental findings. They rarely produce mediastinal compartment syndrome even at considerable sizes and can grow undetected for years. Surgical excision is usually postponed until symptomatic, leading to an increased morbidity associated with surgery, generally requiring cardiopulmonary support. Successful tumor resection is associated with a good prognosis. This case-report illustrates a safe-approach for removal of a large symptomatic retrocardiac mediastinal lipoma, not requiring extracorporeal life support (ECLS) despite cardiac manipulation. This work has been reported in line with the SCARE criteria [1].

## Case presentation

**Figure 1:**
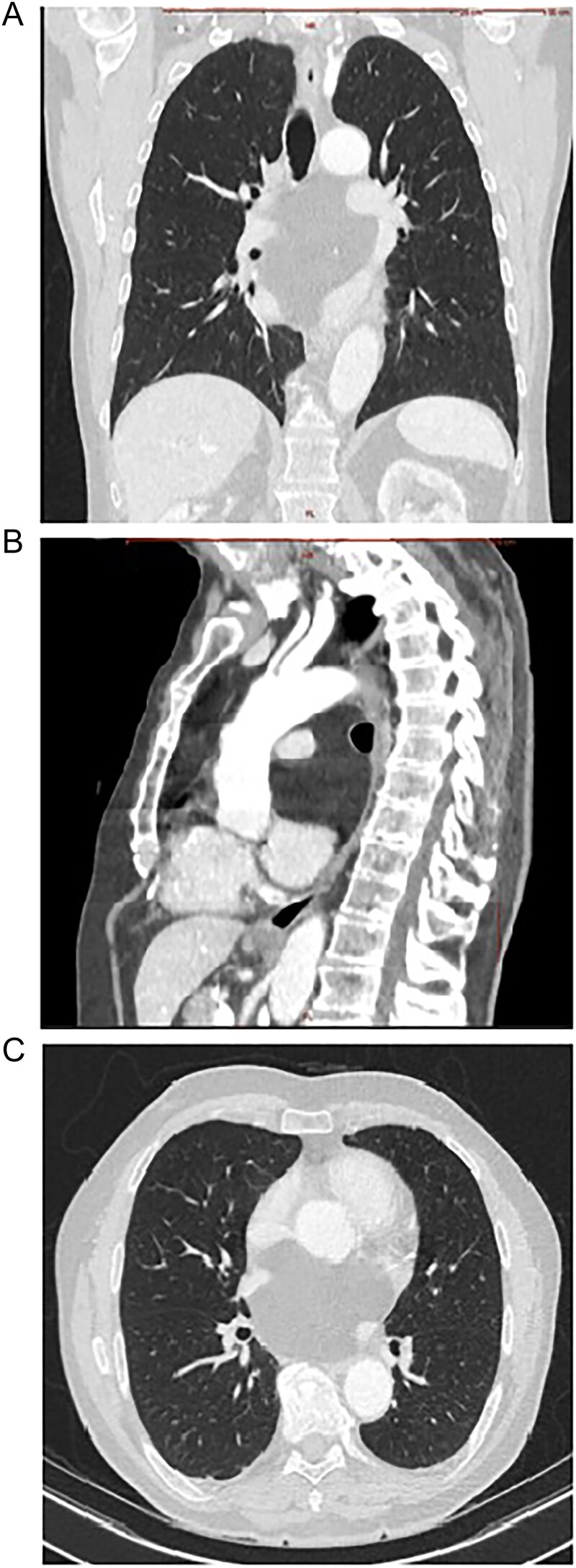
Contrast enhanced CT scan of mediastinum demonstrating the well-defined focal fat-attenuated homogenous lesion. (A) Coronal view, (B) sagittal view, (C)axial view.

A 75-year-old Caucasian man presented with worsened retrosternal pressure, decline in performance and syncope episodes. The patient was investigated 2-years earlier for performance intolerance, revealing a 9 × 8.5 cm mediastinal retrocardiac lesion in a computed tomography (CT)-scan, initially classified as oligosymptomatic. The progredient symptomatology prompted a new CT-scan showing a slight increase in size of the lesion to 8 × 10 cm, compression of the left atrium and pulmonary veins bilaterally (Fig. 1A–C). An echocardiography performed at rest demonstrated a regular cardiac output with no obstruction of flow in the pulmonary veins or arteries. A pre-operative diagnosis of a benign lipoma was suspected. Interdisciplinary concerns about a potentially abrupt and fatal insufficiency during increased exercise led to the referral to surgery.

**Figure 2:**
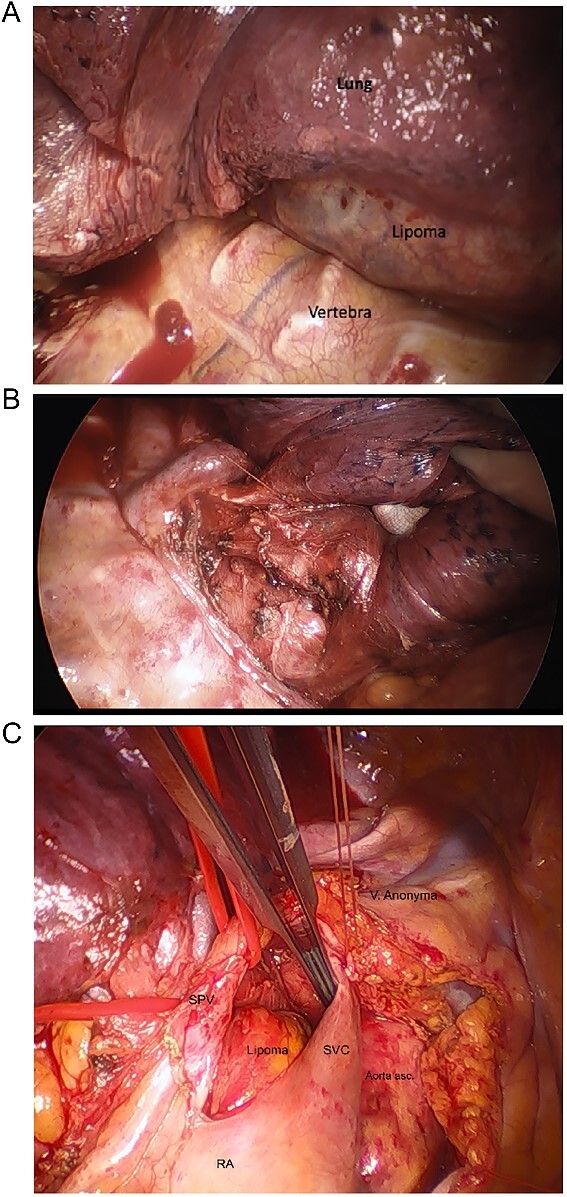
Intraoperative images. (A) After thoracotomy giving access to mediastinum, (B) incision in posterior mediastinal pleura giving access to posterior mediastinum, (C) separating lipoma around SVC, superior pulmonary vein (SPV), ascending aorta, right atrium (RA), hidden pulmonary truncus, and right pulmonary artery.

**Figure 3:**
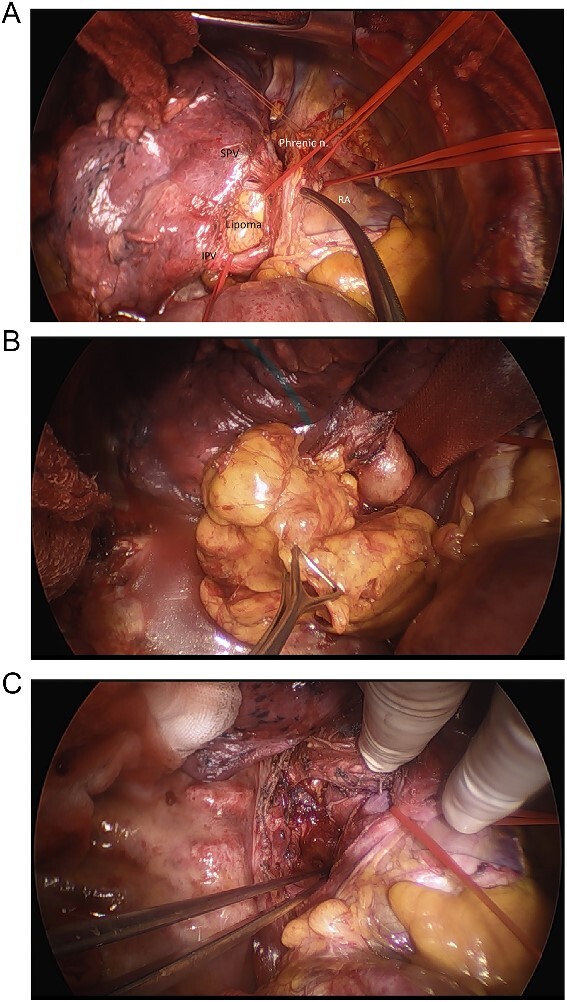
Intraoperative images. (A) Intraoperative view of inferior pulmonary vein (IPV), SPV, phrenic nerve, right atrium, and the lipoma, (B) final removal of the Lipoma from the right incision of the dorsal mediastinum, (C) resection site after removal of lipoma before closure.

The surgical exploration was achieved through a right lateral thoracotomy with a retro- and trans-pericardial approach. The thoracotomy was performed in the fifth intercostal space with division of the fifth rib in the cartilaginous portion with subsequent severing of the pulmonary ligament (Fig. 2A). Following circumferential dissection of the posterior pulmonary hilum the encapsulated soft tumor, reminiscent of a lipoma, became visible in the posterior mediastinum. The inferior pulmonary vein and the left atrium were intricately grown together with the tumor capsule, spanning from an extra- to intrapericardial location which required a meticulous dissection (Fig. 2B, C). Additionally, a 5-cm-long pericardial opening lateral to the phrenic nerve was necessary for complete resection (Fig. 3A). Free dissection of the superior vena cava (SVC) and azygos vein in a dorsal direction was performed to detach the tumor capsule from the retrocaval space and right atrium. An additional 8 cm long longitudinal opening of the pericardium up to the level of the anonymous vein was required. Dissection of the aperture between the SVC and aorta generated a supplementary window to further dissect the tumor capsule dorsally from these structures, the pulmonary artery and the remaining retro-pericardial area. Finally, with ventral mobilization of the heart the tumor resection could be completed (Fig. 3B, C). Upon re-ventilation, the lungs promptly expanded to optimal capacity.

The tumor was successfully resected en bloc in its capsule without any intraoperative complications. The total operation time was 185 min with a total blood loss of <250 ml. Stand-by ECLS was present throughout the procedure in case of hemodynamic insufficiency during cardiac manipulations or bleeding, but its use was not required at any point. The postoperative course was uneventful and 4-h after the operation the patient could be extubated in the intensive-care unit (ICU). 24-h post-surgery the patient could be transferred from the ICU to a regular ward and the chest-drain could be removed after 4 days. The subsequent x-ray revealed distended lungs bilaterally with an unremarkable mediastinal silhouette and the patient was discharged from the hospital in a good general condition on Day 7 post-op. The following day, the patient experienced an episode of atrial fibrillation for which he sought medical attention in his local hospital. In the 12-month control the patient was completely free of symptoms and in a good general condition.

### Histology

**Figure 4:**
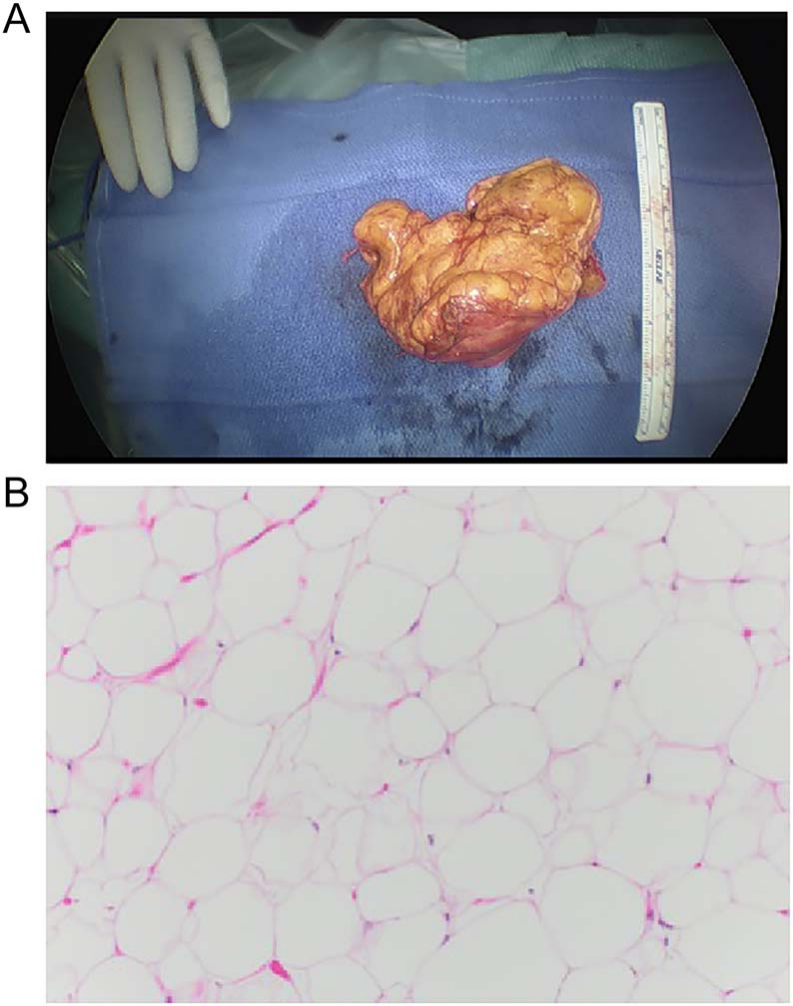
Macro- and microscopic findings. (A) Gross appearance of the lesion, (B) histological examination of the lesion, composed of abundant mature adipose cells with adipocytes exhibiting uniformity in both size and shape. 10× magnification, H&E stain.

Gross appearance showed a lobulated lesion with distinct yellow-fatty-tissue resembling a lipoma. It had a soft texture with a smooth surface covered by a thin shiny membrane. The tumor measured 10 × 8.5 cm and had a formalin-fixed weight of 172 g (Fig. 4A). The microscopic examination showed an encapsulated tumor composed of abundant mature adipose cells with adipocytes that exhibited uniformity in both size and shape (Fig. 4B). There was no detection of lipoblasts, increased mitotic rate, or zones of necrosis indicating malignancy. The definitive pathological examination was a mature retrocardiac mediastinal lipoma without any evidence of malignancy.

## Discussion

Lipomas are the most frequently encountered soft-tissue neoplasm in adults and are rarely situated in the mediastinum [2]. In this case, large mediastinal tumors occur mostly in the anterior portion and represent 1.6–2.3% of all primary mediastinal tumors [3]. A lipomatous lesion of the mediastinum tends to grow slowly and is always classified as benign except those causing mediastinal compartment syndrome. These lesions should be regarded as clinically malignant and treated with en bloc excision [4].

Liposarcomas are a diverse group of malignant soft-tissue tumors. Well-differentiated liposarcomas (WDLS) can be difficult to discern from a benign lipoma on imaging. In a study [O’Donnell *et al*.], experienced musculoskeletal-radiologists and orthopedic-oncologists were able to differentiate between lipomas and WDLS in imaging in 69% of cases [5]. WDLS are associated with amplification of chromosome segment 12q13-15, which carries the oncogenes MDM_2_, CDK_4_, and HMGA_2_ [6]. These amplifications are not seen in lipomas and its presence helps to differentiate them from WDLS [7]. A histological exploration is therefore recommended in the pre-operative phase to differentiate these, especially in surgeries where an en bloc resection can’t be guaranteed. In our case, the suspected pre-operative diagnosis of lipoma was based on imaging in conjunction with the slow progression of the lesion over time. A fluorescence *in situ* hybridization to detect genetic abnormalities involving MDM_2_ to rule out a malignant WDLS should have been performed.

Local recurrence of intrathoracic and mediastinal lipomas is uncommon following complete resection [7]. Generally, surgery is delayed until the lipoma is large, probably because of the morbidity associated with surgery under cardiopulmonary support. This case illustrated an original interdisciplinary, less invasive and safe approach with a good patient outcome. Such an approach allows to treat patients earlier before they develop life-threatening symptoms. Stand-by ECLS is an important pre-cautionary to have available during surgery, but may not be required.
